# Investigating the Efficiency of a Heat Recovery–Storage System Using Heat Pipes and Phase Change Materials

**DOI:** 10.3390/ma16062382

**Published:** 2023-03-16

**Authors:** Robert Stefan Vizitiu, Andrei Burlacu, Chérifa Abid, Marius Costel Balan, Stefanica Eliza Vizitiu, Marius Branoaea, Nicoleta Elena Kaba

**Affiliations:** 1 Faculty of Civil Engineering and Building Services, “Gheorghe Asachi” Technical University of Iasi, 700050 Iasi, Romania; 2 Aix-Marseille Université, CNRS, IUSTI, 13453 Marseille, France; 3 Faculty of Civil Engineering, Politehnica University of Timisoara, 300223 Timișoara, Romania

**Keywords:** heat recovery, heat storage, heat pipe, PCM

## Abstract

This study presents an experimental and numerical investigation into the efficiency of a two-stage heat recovery–storage system for reducing the thermal energy losses in the industry. The system is designed to recover and store waste thermal energy from residual fluids using heat pipes for recovery and an environmentally friendly phase change material for heat storage. Experimental investigation was conducted using water as the primary agent and varying the temperature between 60 °C, 65 °C, and 70 °C at a constant flow rate of 24 L/min. The secondary agent, also water, was used at an initial temperature of 10 °C and the flow rate was varied between 1 L/min, 2 L/min, and 3 L/min. The results show that the system had a peak efficiency of 78.1% and was able to recover a significant amount of thermal energy. This study demonstrates the potential of this system to reduce the thermal energy losses in the industry and highlight the importance of further research and development in this field, as the industry is responsible for approximately 14% of the total thermal energy losses and finding efficient ways to recover and store waste thermal energy is crucial to achieving sustainable energy consumption.

## 1. Introduction

The negative impact of fossil fuels on the climate is well known and humanity is seeking non-polluting, renewable resources to replace them. The Paris Agreement of 2017 aims to keep the global temperature rise below 2 °C by the end of the century, which would require USD 3.5 trillion in annual investments in renewable and sustainable energy by 2050, according to a study by the International Energy Agency and International Renewable Energy Agency. In 2015, USD 1.8 trillion was invested in energy, with 12% going towards energy efficiency [1].

Heat recovery is a technique that has the potential to contribute to the reduction in greenhouse gas emissions. The process involves the capture and conversion of residual thermal energy into useful forms of energy, such as thermal, electrical, or mechanical energy. The most common forms of residual thermal energy are steam, hot air, or hot water, and the efficiency of the heat recovery process increases with the temperature of the residual heat. There are various heat recovery technologies available, including energy recovery heat exchangers, such as waste heat recovery units. Among these, heat pipe heat exchangers are considered particularly efficient due to the high thermal conductivity of heat pipes, which allows for a minimal temperature drop during heat transfer over long distances.

A number of studies have been conducted to evaluate the various types of heat recovery systems, primarily utilizing residual heat from industrial sources. The sources of energy that can be recovered in this manner include the heat generated from industrial equipment, heat generated from combustion processes, and the heat lost through radiation, conduction, or convection during industrial processes [2].

Jouhara et al. [3] designed and studied a radiative heat recovery system for recovering waste heat radiated during the steel manufacturing process. Another study, conducted by Delpech et al. [4], examined a heat recovery system that captures waste heat from the cooling of ceramic parts. The results of this study indicate that, at temperatures below 200 °C, the heat transfer is primarily convective, while at higher temperatures, the heat transfer is a combination of radiation and convection. Other studies have also been conducted on heat pipe heat recovery systems utilizing flue gases [5] and hot air [6,7] as primary sources. The waste heat recovery from steam is a well-established method and there have been studies investigating the use of steam generated from nuclear power plants as a primary source of seawater desalination [8].

The heat recovery process can be further optimized by incorporating latent heat storage systems. These systems consist of three main components: a storage medium, typically a phase change material, a heat transfer mechanism, and a containment system [9]. However, one of the main limitations of phase change materials is their low thermal conductivity, which has led to numerous studies aiming to improving their performance [10,11,12,13].

There are some experiments combining heat pipe technology with phase change materials [14,15]. A study [16] performed in 2017 by Amini et al. investigated a heat recovery system that uses steam as a primary agent and a salt hydrate PCM which melts at 89 °C as the heat storage medium. The results showed the good efficiency of the systems but it can be improved by increasing the thermal conductivity of the PCM.

Fan et al. [17] investigated a two-stage latent thermal energy storage tank that is integrated with an air purification pilot plant. A test was then carried out under actual operating conditions. The tank has units that are filled with commercial paraffin with a melting point of 70 °C and 48 °C, respectively. For a better heat transfer performance, heat pipes are combined with vertical fins for this research. According to the results, adding a latent thermal energy storage tank to an air separation system allows for a 51,369.5 kJ heat savings during a charging–discharging session. The waste heat requirement of 40 °C allowed for the recovery of 64.7% of the excess heat in the exhaust air and annually, 25,784.3 kg less CO_2_ is emitted. In contrast with previous known latent thermal energy storage for industrial purposes, the latent thermal energy storage exhibits great results.

In another study [18] from 2015, a high-temperature latent heat thermal energy storage system assisted by finned heat pipes was subjected to numerical simulations, to check the charging process of PCM with a different configuration of embedded heat pipes. It was found that the bigger the number of heat pipes, the smaller the thermal resistance within the system, resulting a faster charging process.

This previous study is relevant to the recent research, as the use of finned storage units was also explored by [19], as a method to enhance the thermal performance of a vertically configured-cylindrical copper LHTES in combination with nano-enhanced PCM (NEPCM) and both outer and inner longitudinal fins. The study’s outcomes demonstrate that the inclusion of six longitudinal fins on the internal and external surfaces of the LHTES wall leads to a notable decrease in the duration needed for discharging and charging by 71% and 62%, correspondingly, compared to the pure PCM reference condition lacking fins.

In their study, Nie et al. [20] also investigated the aspects of successively charging and discharging the phase change material in a thermal energy storage unit with fins. According to the results, it was observed that the heat transmission was enhanced when using a straight fin design, particularly when there are fewer fins used and when the thermal conductivity of the outer tube material is lower. Additionally, it was discovered that, when continuously charging and discharging, heat transfer was improved by using longer fins rather than shorter fins, while the fin volume remained the same.

In-depth experimental and computational investigation by Ruiz et al. [21] resulted in the determination of the optimum design for a water heat recovery and storage unit based on phase change material plates. The phase change material storage unit is designed to store approximately 75% of the thermal energy during the heat charging phase and some of it is recovered to provide approximately 50% of the thermal energy needed for the subsequent process. The performance of heat transfer for the latent thermal energy storage unit appeared to be strongly linked to the working parameters.

In order to enhance the efficiency of latent heat thermal energy storage (LHTES) with embedded heat pipes, Ladekar et al. [22] experimentally investigated the charging and discharging process in the case of using a heat pipe and a copper pipe. The results show that the capability to store heat for the charging process phase increases when majoring the volume flow rate, and early heat extraction when discharging becomes achievable. The charging and discharging of latent LHTES with integrated heat pipes performed well during testing with a flow rate of 10 L/min. When compared to systems with copper pipes, LHTES with integrated heat pipes has greater and more consistent efficiency and efficacy. It was also concluded that, as the volume flow drops, the overall melting time of the phase-changing material reduces.

The novelty of this research lies in the combination of gravitational heat pipes and phase change materials in a heat recovery–storage system (HRSS) for recovering and storing waste thermal energy from residual fluids. The system utilizes gravitational heat pipes to recover the low-temperature residual heat and store it as latent heat within a phase change material.

Previous research has separately focused on either the use of heat pipes or phase change materials, indicating the lack of experimental studies using both the quick heat transfer ability of heat pipes and the latent heat storage property of phase change materials. Therefore, it is essential to give more attention to the experimental part in order to reveal methods for improving the heat transfer performance of phase change materials and heat pipes [23].

This study proposes a new approach that combines both technologies to increase the efficiency of the heat recovery process and store thermal energy for future use. Heat pipes are used in the device’s modular design to increase the efficiency of the heat recovery process, and phase change materials are used to store the thermal energy. The apparatus can recover the waste thermal energy from used water at low and medium temperatures. The thermal energy can then be transformed into additional thermal energy and used to pre-heat or heat water for domestic use or other heating systems.

This research effort seeks to examine the potential of the suggested HRSS in recovering and storing waste thermal energy from low- to medium-temperature residual fluids. The study intends to provide experimental proof of the HRSS’ efficiency and highlight the technology’s potential benefits in reducing thermal energy losses and achieving sustainable energy management.

In the following sections of this paper, there are descriptions of the design of the equipment and its testing parameters, followed by the presentation of results and conclusions.

## 2. Materials and Methods

### 2.1. The Design of the Equipment

The two-stage heat recovery–storage system consists of the connection of a water-to-water heat exchanger (heat exchanger A) to a water-to-water heat exchanger with integrated PCM (heat exchanger B). Heat exchanger A was previously tested as a stand-alone heat recovery system, revealing a high efficiency of up to 76.7% in recovering low- and medium-temperature thermal energy [24].

**Figure 1:**
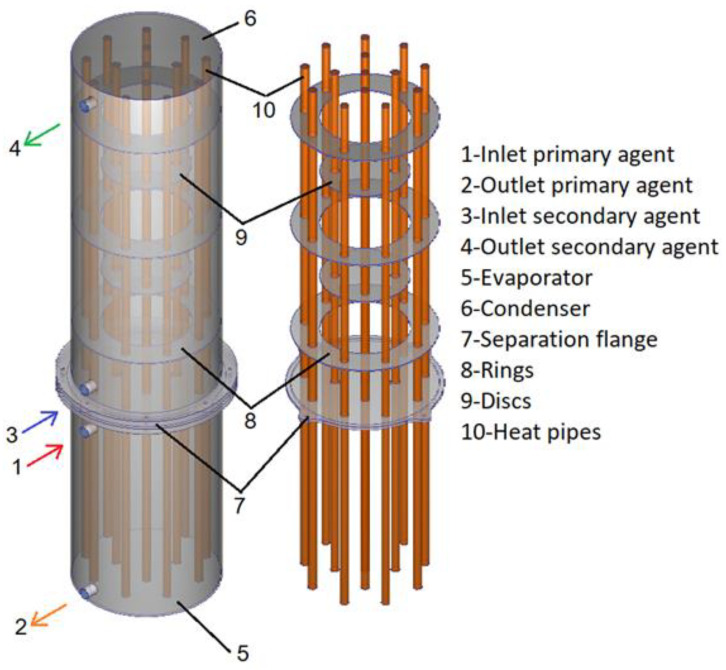
The design of the water–water heat exchanger (heat exchanger A).

The design of heat exchanger A is presented in Figure 1. The heat exchanger consists of two main areas called the evaporator and condenser, separated by a sealing flange. Fourteen heat pipes are inserted transversely inside the equipment with the role of extracting thermal energy from the primary agent inside the evaporator and transferring it to the secondary agent inside the condenser. Inside the condenser, there are three rings and two discs, creating a path for the secondary agent. The evaporator, condenser, and separation flange are made of stainless steel and the rings, discs, and heat pipes are made of copper. The working fluid of the heat pipes is distilled water with a fill ratio of 25%.

The design of heat exchanger B is similar to the design of the heat exchanger, but the major difference is given by the fact that the secondary agent is directed through the condenser by a coil and the volume of the condenser is filled with a phase change material. The coil is made of copper, has a total length of 3.5 m, and has 7 turns with a diameter of 15 cm. The phase change material is coconut oil, and the necessary quantity to fill the condenser is 26.4 kg. The thermophysical properties of these phase change materials are presented in Table 1 [25].

**Figure 2:**
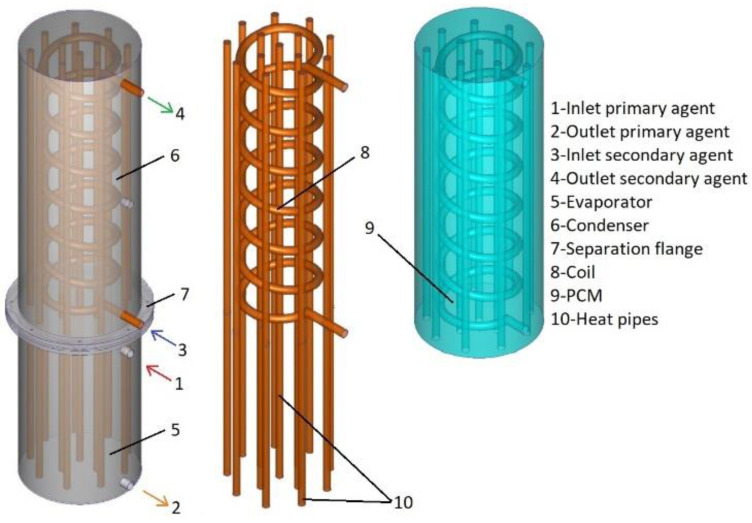
The design of the PCM heat exchanger (heat exchanger B).

Figure 2 presents the design of heat exchanger B in 3D.

The constructive details of the two heat exchangers can be viewed in Table 2.

### 2.2. Experimental Setup

The thermal agent from which heat is recovered (primary agent) is water, heated by an 8 kW electric heater. The electric heater is connected to the heat recovery–storage system through flexible stainless-steel pipes. The circuit is equipped with a valve for filling/draining the evaporator and the primary circuit and a probe sheath for measuring the temperature of the primary agent.

**Figure 3:**
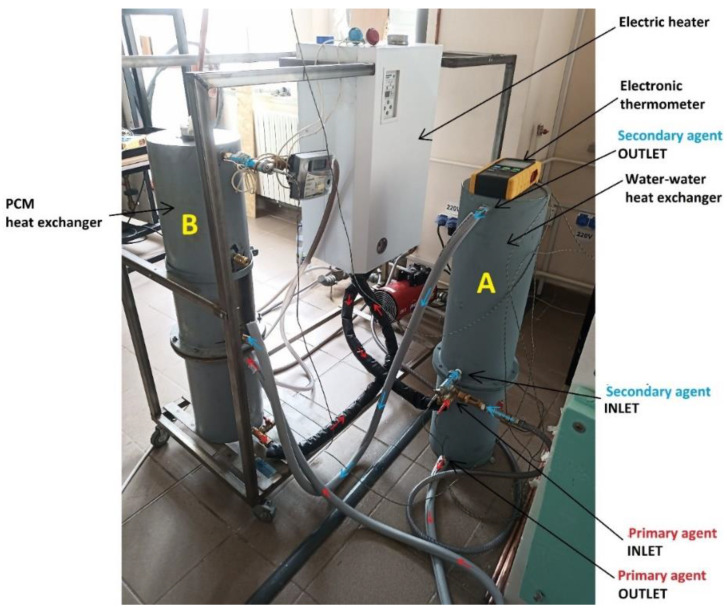
Photo of the experimental stand.

The circuit of the secondary agent is directly connected to the water supply network of the laboratory. Two probe sheaths were installed at the inlet of condenser A and at the outlet of condenser B, connected to an electronic thermometer type LT BTM-4208SD which has a precision of ±0.4%. A thermal energy meter of type ELTERM CF 55 is also tracking the volume flow rate and the temperature of the secondary agent at the outlet of condenser B. Figure 3 presents a photo of the experimental stand from the laboratory.

In this study, an electric heater is used to heat the primary agent, which flows through the evaporator of two heat exchangers, A and B. The heat pipes are used to extract thermal energy from the primary agent and transfer it to a secondary agent flowing through heat exchanger A and a phase change material (PCM) volume in heat exchanger B. The heated secondary agent, which exits condenser A, passes through the coil of condenser B, where it extracts additional temperature from the volume of coconut oil. Four sensors are used to measure the temperature of the primary and secondary agents, as well as the PCM. Sensor CH1 tracks the temperature of the primary agent, while sensors CH2 and CH3 track the temperature of the secondary agent at the inlet and outlet of condenser, respectively. Sensor CH4 tracks the temperature of the PCM.

**Figure 4:**
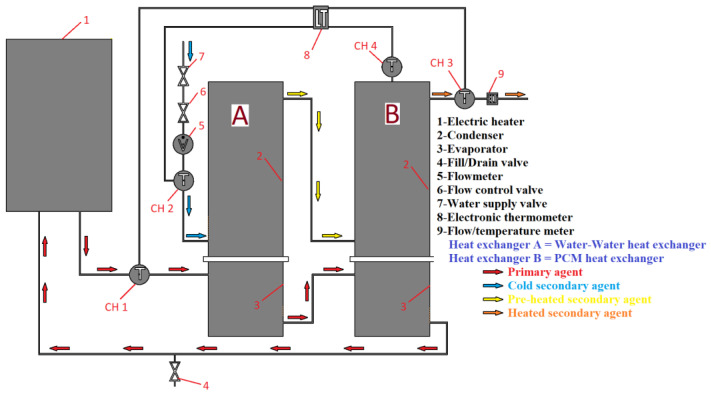
Schematic diagram of the 2-stage heat recovery–storage system.

Figure 4 presents a schematic diagram of the experimental two-stage heat recovery–storage system.

### 2.3. Tests Performed

To test the efficiency of the equipment, a series of tests were performed with variations in the temperature of the primary agent and the volume flow rate of the secondary agent. The parameters are presented in Table 3. The volume flow rate of the primary agent was kept constant for all cases by the pump of the electric heater, and the temperature of the secondary agent was kept at 16 °C.

**Figure 5:**
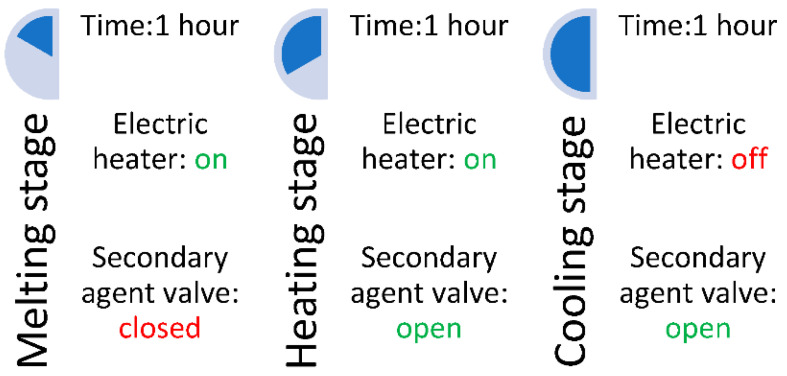
The stages of the tests.

Each test lasted 3 h, being staged according to Figure 5. The staging followed the behavior of the heat recovery–storage system and the ability to store the thermal energy of the coconut oil. In the first hour, the electric heater operated constantly to melt the coconut oil and transfer heat to the volume of the secondary agent in unit A. After one hour, the valve of the secondary agent was opened at the volume flow required by the test, thus starting its circulation through the heat exchangers. After the first two hours of operation, the electric heater was turned off, starting the cooling stage where the secondary agent absorbed the heat stored in the volume of phase-changing material.

This staging was aimed at the equipment’s ability to recover heat in 2 scenarios: if the primary agent has an uninterrupted supply and in the case of an intermittent supply with the primary agent.

### 2.4. Numerical Simulations

For results with improved accuracy, the heat recovery system was subjected to numerical simulations and the results were compared to the experimental findings. The equipment was created in 3D with the Autodesk Inventor Professional 2018 software, and imported into the Autodesk Simulation CFD 2018 environment.

The first step is to assign a material to each component. The evaporator, the condenser, and the separation flanges are made of stainless steel, while the heat pipes and the coil are made of copper and the primary and secondary agents are water. Coconut oil does not exist in the program database, so a new material with its thermal properties was created.

The boundary conditions used are the same as in the experimental tests. The volume flow rate of the primary agent was set at 24 L/min and the temperature was set specifically for each test. The temperature of the secondary agent was set at 10 °C and the volume flow rates were set according to each scenario (Table 3).

To select the proper flow regime, the Reynolds number was calculated for each case. The results are centralized in Table 4. When the volume flow rate of the secondary agent is 1 L/min or 2 L/min, the Reynolds number is smaller than 2300, resulting in a laminar flow. When the volume flow rate is 3 L/min, the Reynolds number has a value of approximately 2460 which means that there is a transition regime.

**Figure 6:**
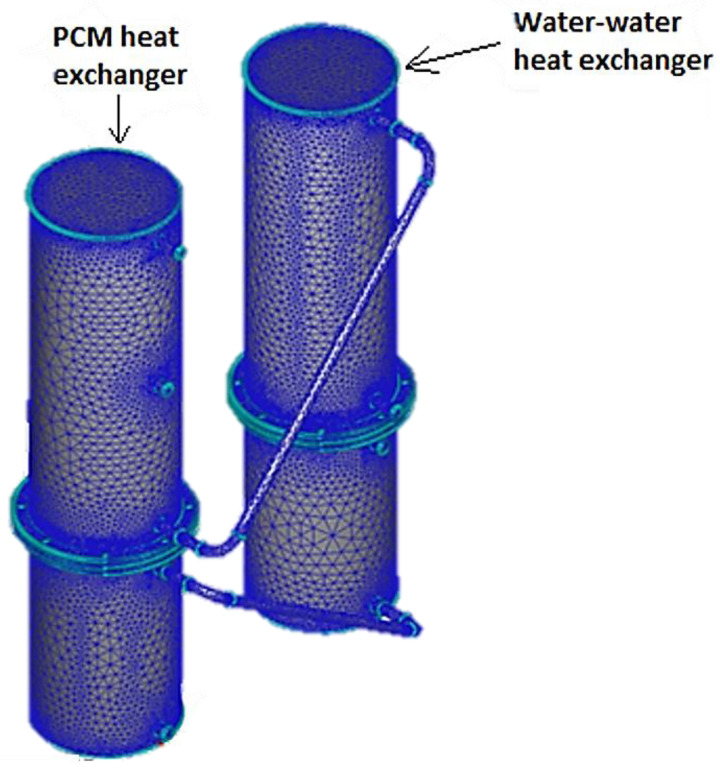
Meshing—2-stage heat recovery–storage system.

After applying the boundary conditions, the 3D model was discretized into a network of nodes. For the first simulation, the geometry was discretized into a network that consists of 1 million nodes. Then, the number of nodes was increased by 1 million for each simulation until the mesh reached 7 million nodes. After 5 million nodes, the results were very similar. The higher the number of discretization elements, the greater the processing power and more time was needed, so the number of elements for the calculation of the solution was optimized according to the criteria and the tests performed. The resulting mesh had 5.2 million nodes (Figure 6).

Experimental testing was conducted in the laboratory in order to evaluate the performance of the 2-stage heat recovery–storage system. The equipment was tested using several temperatures for the primary agent to check whether the heat pipes are capable of efficiently recovering heat in different scenarios, and different volume flow rates for the primary agent to check the quantity of heat recovered in different instances. Additionally, to check the efficiency of the heat storage, the measurements of temperatures were made in three different stages: a melting stage, a heating stage, and a cooling stage. A detailed description of these stages is given in Section 2.3.

The last step was the calculation of the maximum quantity of heat contained by the primary agent and the quantity of heat recovered by the secondary agent. By comparing these, the efficiency of the equipment can be established for each test.

## 3. Results and Discussions

**Figure 7:**
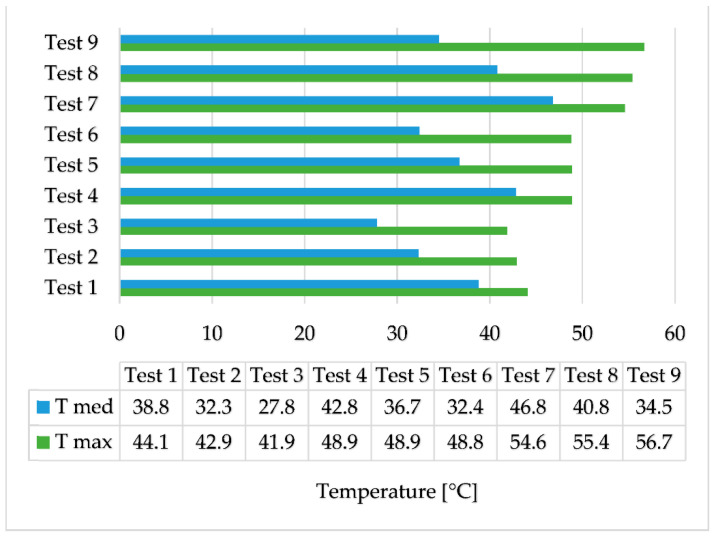
Average and maximum temperatures obtained.

The results of the experimental tests are centralized in Figure 7. T max represents the maximum temperature reached by the two-stage recovery system and T med represents the average temperature of the secondary agent at the outlet of the PCM heat exchanger during the heating stage.

**Figure 8:**
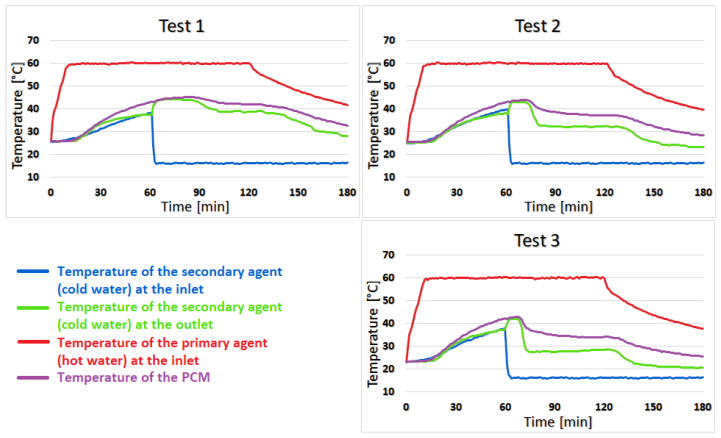
The results for tests 1, 2, and 3.

In test 1, during the first 27 min of the heating stage, the difference between the temperature of the coconut oil and the temperature of the secondary agent varies between 0 and 2 °C. During this period, this difference increased to 3–5 °C, which is maintained until the end of the test. For test 2, the difference of 1–2 °C was only in the first 10 min of the heating stage, and later, this difference increased to 5–7 °C and remains constant. In test 3, the difference of 1–2 °C was shortened to 8 min but still reached 5–7 °C by the end of the test, as shown in Figure 8.

**Figure 9:**
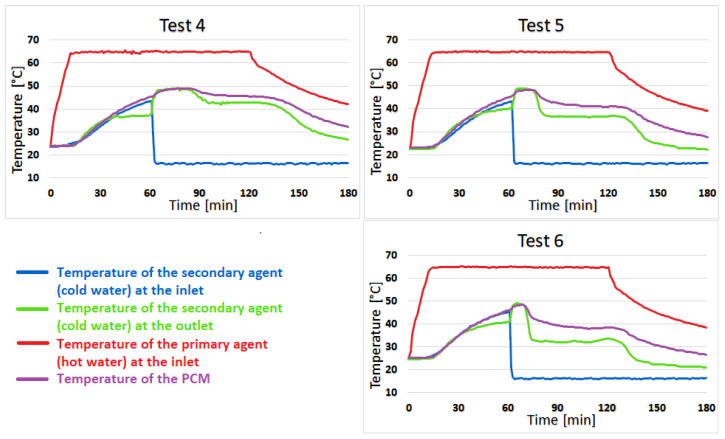
The results for tests 4, 5, and 6.

Test 4 shows almost no temperature difference between coconut oil and the secondary agent for the first 23 min of the heating stage, but the difference increases to 2–3 °C for the next 52 min and towards the second half of the cooling stage, this difference becomes 4–6 °C. The similarity of the temperature of coconut oil to that of the secondary agent lasts 13 min in test 5 with a difference of 0–2 °C. The difference increases quite suddenly at 5–8 °C and is maintained until the end of the test. For test 6, the duration of the 0–2 °C variation is shortened to only 7 min, and the temperature difference between the PCM and the secondary agent sharply increases to 5–9 °C, as can be seen in Figure 9.

**Figure 10:**
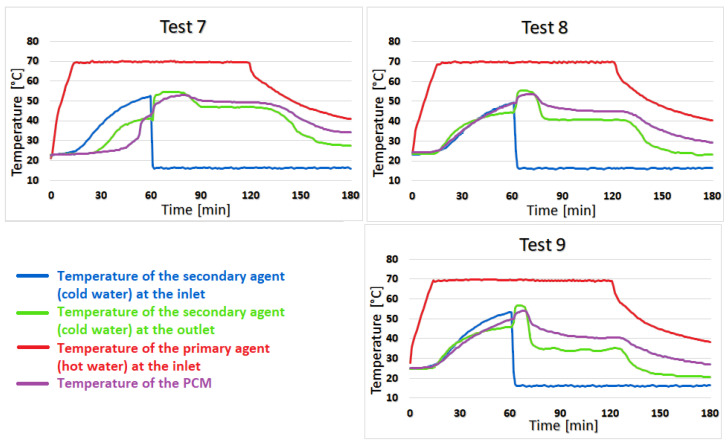
The results for tests 7, 8, and 9.

Test 7 shows the best results of the two-stage recovery system, with an average temperature of the secondary agent during the heating stage of 46.8 °C. The temperature of the secondary agent is higher than the temperature of the coconut oil in the first 20 min of the heating stage. In the last 40 min of this stage, the temperature at the outlet drops below the temperature of the phase change material by 3 °C. The difference then increases to 4–7 °C in the cooling stage and is maintained until the end of the test. Additionally, in tests 8 and 9, the temperature of the secondary agent is higher than that of coconut oil for a short time, but then decreases at the same rate as it, as can be seen in Figure 10.

**Figure 11:**
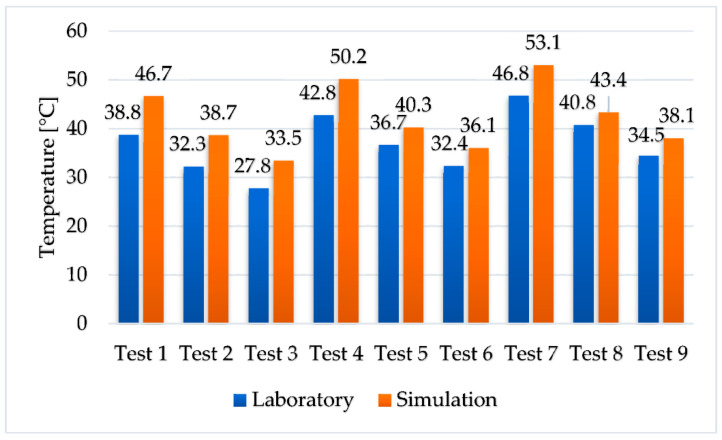
Comparison between the experimental and numerical results.

The temperatures of the secondary agent recorded at the outlet of the system were compared to the average temperatures from the experimental results and they are presented in Figure 11.

Since the simulations do not take into account the convective heat losses from the laboratory experiment, the temperature in the experimental results is lower than in the numerical simulations, with values between 3 °C and 8 °C. Since the temperature discrepancies between the experimental results and the simulation results are negligible, it can be concluded that the experimental results are reliable.

**Figure 12:**
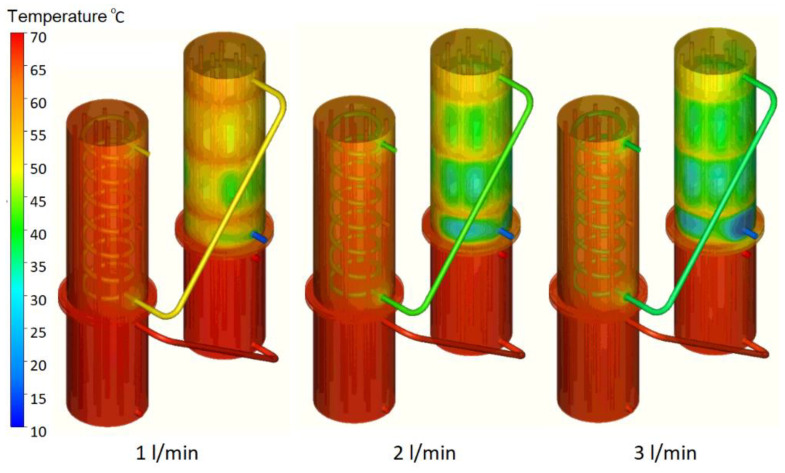
Three-dimensional temperature contours for tests 7, 8, and 9.

In Figure 12, some 3D temperature contours extracted from the results of the numerical simulations are presented for tests 7, 8, and 9 at the end of the cooling stage. The temperature of the secondary agent increases between 3 °C and 6 °C from the outlet of the first condenser to the outlet of the system but the main advantage of the two-stage heat recovery system is the storage of thermal energy, which cannot be tracked in the numerical simulations due to software limitations.

**Figure 13:**
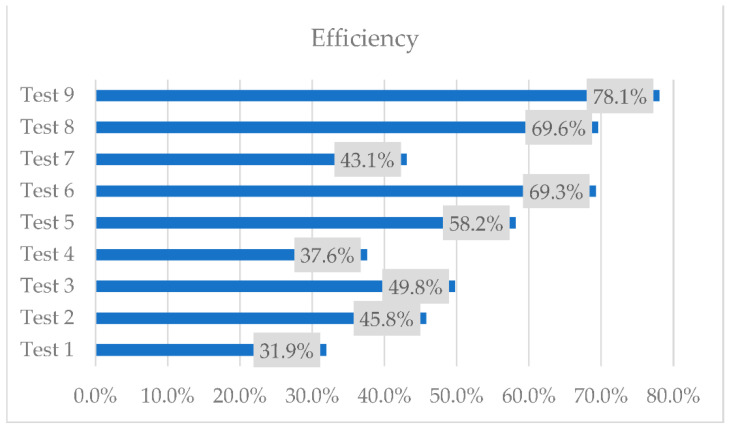
The efficiency of the 2-stage heat recovery–storage system.

The heat quantity in the primary agent was calculated as the product of the mass flow rate, the specific heat capacity of water, and the temperature difference between evaporator A’s inlet and evaporator B’s outlet. The heat quantity of the secondary agent was determined in a comparable manner, using the secondary agent’s mass flow rate, the specific heat capacity of water, and the temperature difference between T med (Figure 7) for each scenario and the cold secondary agent’s temperature of 16 °C (Table 4). According to Equation (1), the efficiency of the two-stage heat recovery–storage system was calculated as a fraction of the heat recovered by the secondary agent and the maximum amount of heat produced by the primary agent. The results are shown in Figure 13.

ϵ = Q_2_/Q_1_ (1)

The highest efficiency was obtained in test 9 when the volume flow rate of the secondary agent is 3 L/min and the temperature of the primary agent is 70 °C. Although the highest temperatures of the secondary agent were obtained with the smallest volume flow rate, the quantity of heat recovered is smaller.

## 4. Conclusions

An experimental investigation followed by numerical simulations was carried out on a two-stage heat recovery–storage system. From the experimental results, the highest temperature recorded was when the volume flow rate of the secondary agent was the lowest and the temperature of the primary agent was the highest, at 70 °C, with an average temperature of the secondary agent of 46.8 °C. However, the highest efficiency of the equipment regarding the quantity of the recovered heat was in test 9, with an efficiency of 78.1%.

The ability of the equipment to store heat was observed in all nine tests conducted. During the cooling stage, when the heat source is shut down, the secondary agent still keeps a higher temperature at the outlet of the equipment compared to the one at the inlet by 11.5 °C–4.1 °C, depending on its volume flow rate.

Compared to a common heat recovery system, the two-stage heat recovery–storage system comes with a heat storage capability, which makes the heat recovery process more efficient, especially when the heat source is intermittent. The organic phase change material used for heat storage is a plant-based product, which makes it environmentally friendly.

Another important advantage is the ability to recover heat passively through the gravitational heat pipes, which means that there is no additional pumping system required. The two-stage heat recovery–storage system also has a low cost of production and maintenance compared to common heat exchangers. The two thermal agents are completely separated since the mean of transporting the heat are the heat pipes, which means there are very small chances of accidental mixing. Additionally, if one of the heat pipes malfunctions, the equipment can still function at a slower pace until the heat pipe is replaced, and thus, the maintenance is simple.

## Figures and Tables

**Table 1:** Thermophysical properties of coconut oil [24].

Mean specific heat of the solid	3.2 kJ/kg × K
Mean specific heat of the liquid	4.1 kJ/kg × K
Heat of fusion	249 kJ/kg
Melting temperature	35 °C

**Table 2:** Constructive details of the components.

Component	Height/Length (m)	Diameter (m)
Evaporator	0.395	0.250
Condenser	0.640	0.250
Separation flange	0.005	0.300
Discs	0.005	0.150
Rings	0.005	0.246
Heat pipes	1.000	0.015
Coil	3.500	0.015

**Table 3:** Parameters of the experimental tests.

	Primary Agent		Secondary Agent	
	Temperature (°C)	Volume Flow Rate (L/min)	Temperature (°C)	Volume Flow Rate (L/min)
Test 1	60	24	16	1
Test 2	60		16	2
Test 3	60		16	3
Test 4	65		16	1
Test 5	65		16	2
Test 6	65		16	3
Test 7	70		16	1
Test 8	70		16	2
Test 9	70		16	3

**Table 4:** Reynolds number.

Secondary Agent				
	Temperature (°C)	Volume Flow Rate (L/min)	Reynolds	Regime
Test 1	16	1	819.8	Laminar
Test 2	16	2	1640.4	Laminar
Test 3	16	3	2461.3	Transition
Test 4	16	1	820.0	Laminar
Test 5	16	2	1640.9	Laminar
Test 6	16	3	2460.1	Transition
Test 7	16	1	820.5	Laminar
Test 8	16	2	1640.6	Laminar
Test 9	16	3	2460.9	Transition

## Data Availability

Not applicable.

## Nomenclature

PCM	Phase change material
NEPCM	Nano-enhanced phase change material
HRSS	Heat recovery–storage system
LHTES	Latent heat thermal energy storage
T max	Maximum temperature
T med	Average temperature
ϵ	Efficiency of the equipment
Q_1_	Quantity of heat of the primary agent
Q_2_	Quantity of heat of the secondary agent
